# Automatic Semantic Segmentation of Brain Gliomas from MRI Images Using a Deep Cascaded Neural Network

**DOI:** 10.1155/2018/4940593

**Published:** 2018-03-19

**Authors:** Shaoguo Cui, Lei Mao, Jingfeng Jiang, Chang Liu, Shuyu Xiong

**Affiliations:** ^1^College of Computer Science and Engineering, Chongqing University of Technology, Chongqing 400054, China; ^2^Medical Physics Department, University of Wisconsin, Madison, WI 53705, USA; ^3^Biomedical Engineering Department, Michigan Technological University, Houghton, MI 49931, USA

## Abstract

Brain tumors can appear anywhere in the brain and have vastly different sizes and morphology. Additionally, these tumors are often diffused and poorly contrasted. Consequently, the segmentation of brain tumor and intratumor subregions using magnetic resonance imaging (MRI) data with minimal human interventions remains a challenging task. In this paper, we present a novel fully automatic segmentation method from MRI data containing *in vivo* brain gliomas. This approach can not only localize the entire tumor region but can also accurately segment the intratumor structure. The proposed work was based on a cascaded deep learning convolutional neural network consisting of two subnetworks: (1) a tumor localization network (TLN) and (2) an intratumor classification network (ITCN). The TLN, a fully convolutional network (FCN) in conjunction with the transfer learning technology, was used to first process MRI data. The goal of the first subnetwork was to define the tumor region from an MRI slice. Then, the ITCN was used to label the defined tumor region into multiple subregions. Particularly, ITCN exploited a convolutional neural network (CNN) with deeper architecture and smaller kernel. The proposed approach was validated on multimodal brain tumor segmentation (BRATS 2015) datasets, which contain 220 high-grade glioma (HGG) and 54 low-grade glioma (LGG) cases. Dice similarity coefficient (DSC), positive predictive value (PPV), and sensitivity were used as evaluation metrics. Our experimental results indicated that our method could obtain the promising segmentation results and had a faster segmentation speed. More specifically, the proposed method obtained comparable and overall better DSC values (0.89, 0.77, and 0.80) on the combined (HGG + LGG) testing set, as compared to other methods reported in the literature. Additionally, the proposed approach was able to complete a segmentation task at a rate of 1.54 seconds per slice.

## 1. Introduction

Although brain cancers are less prevalent, they are very lethal. Among them, gliomas are the most common brain tumors. They can be graded into low-grade gliomas (LGG) and high-grade gliomas (HGG), with the latter being more aggressive and infiltrative than the former [1]. A glioma is highly invasive because it tends to aggressively grow and could quickly invade the central nervous system (CNS). According to US National Cancer Institute, approximately 18,000 Americans are diagnosed with a glioma every year; many of them die within 14 months [2]. In clinical practice, medical imaging, mainly computed tomography (CT) and magnetic resonance imaging (MRI), has been used to determine (1) the presence of a tumor, (2) the inclusion of peritumoral edema, and (3) the spread into other locations such as the CNS [3].

Compared to CT, MRI or contrast-enhanced MRI becomes the imaging modality of choice for diagnosis and treatment planning in the brain because of its sensitivity and superior image contrast in soft tissues. However, the multiplicity and complexity of the brain tumors under MRI often make tumor recognition and segmentation difficult for radiologists and other clinicians [4]. Consequently, automatic segmentation of heterogeneous tumors can greatly impact the clinical medicine by freeing physicians from the burden of the manual depiction of tumors. Furthermore, if computer algorithms can provide robust and quantitative measurements of tumor depiction, these automated measurements will greatly aid in the clinical management of brain tumors.

In the past few decades, significant research efforts in the computer vision and image processing community have been devoted to developing computer-aided systems that can be used for automated tumor characterization/classification [5–21]. Although some systems were tested and showed good performance, the fully automatic detection and subsequent diagnosis of brain tumors have not been massively used in the clinical settings, thereby indicating that some major developments are still needed [21].

Based on MRI data, our primary goal of this paper was to propose a new fast and accurate computer system that could first localize complete tumor region and then segment the more detailed intratumor structure. Our computer system contained two major steps. First, by leveraging an FCN [22], a tumor location map was first obtained. In the second step, a deep learning ensemble of the CNN was used to classify the tumor region into four subregions: (1) necrosis, (2) edema, (3) nonenhancing tumor, and (4) enhancing tumor. In this study, the performance of the proposed algorithm was assessed in a public database containing 274 cases of *in vivo* gliomas.

The paper is structured as follows: Section 2 presents the related works in the automated brain cancer segmentation. Particularly, attention was given to computer systems based on machine learning. The proposed two-step (cascaded) neural network is described in Section 3. The emphases are on the design methodology and training methods for the performance assessment. In Section 4, results of our numerical experiments are summarized followed by some closing remarks in Section 5.

## 2. Relevant Work and Our Contributions

In recent years, many methods have been proposed to automatically segment brain tumors based on MRI data. These methods can be largely divided into two categories: (1) hand-crafted feature and classifier methods based on traditional machine learning such as support vector machine (SVM) and random forests (RF) [5–13] and (2) fully automatic methods based on deep learning using the CNN [14–21].

Methods in the first category use manually extracted features, and these features are input to classifiers. In other words, once these hand-crafted features are solely determined by human operators, classifiers “weigh” them during the training but cannot modify these features in any way. One significant concern of hand-crafted features stems from the fact that these features could have significant inter- and intrauser variability. A brief summary of these methods can be found in Table 1.

In contrast, methods in the second category can self-learn the feature representations adapted to a specific task from training data. Recently, deep learning neural networks, especially CNNs, are rapidly gaining their popularity in the computer vision community. This trend has certainly been accelerated after the recent record-shattering performance of the CNN in the ImageNet Large-Scale Visual Recognition Challenge (ILSVRC) [23]. Recent deep learning methods for automatic brain tumor segmentation are summarized below in Table 2.

However, the above-mentioned CNN methods were all based on the patch-wise method in which (medical) images were often divided into patches during the training and testing. The advantage of this method was that it could take advantage of the existing classification model of the natural image and solve the problem of the class label imbalance in MRI images. Despite its popularity, operating on image patches was computationally time-consuming. Recalling, given a typical image size (e.g., 256 × 256), a large number of patches (65535) were required as inputs for prediction. Furthermore, this method was not end-to-end and performed the segmentation task by independently classifying the central pixel of a patch, which will result in some errors and need postprocessing. Thus, the expensive computation and postprocessing become the bottleneck of its real-time clinic application.

Recently, Shelhamer et al. [22] presented a novel FCN for semantic segmentation of natural scene images. This model can be trained in an end-to-end manner (also known as pixel-wise). Their results showed that the FCN outperformed the previous methods for semantic segmentation of a natural scene image in performance and speed. Inspired by the work in [22], we proposed a hybrid approach by constructing a deep cascaded neural network.

Our main contribution of this work is to propose a hybrid cascaded neural network for the purpose of segmentation of brain tumors including segmentation of intratumor subregions, from MRI data. This model consists of one FCN and one CNN. This combination enables us to perform pixel semantic predictions by taking advantage of both a pixel-wise method and a patch-wise method. Formally, in this cascaded neural network, an FCN was first used to localize the tumor region from an MRI slice and then a CNN with deeper architecture and smaller kernels was used to classify brain tumor into multiple subregions. This approach can not only obtain the better segmentation accuracy but can also speed the prediction efficiency.

## 3. Methods

### 3.1. Construction of the Deep Cascaded Neural Network

**Figure 1:**
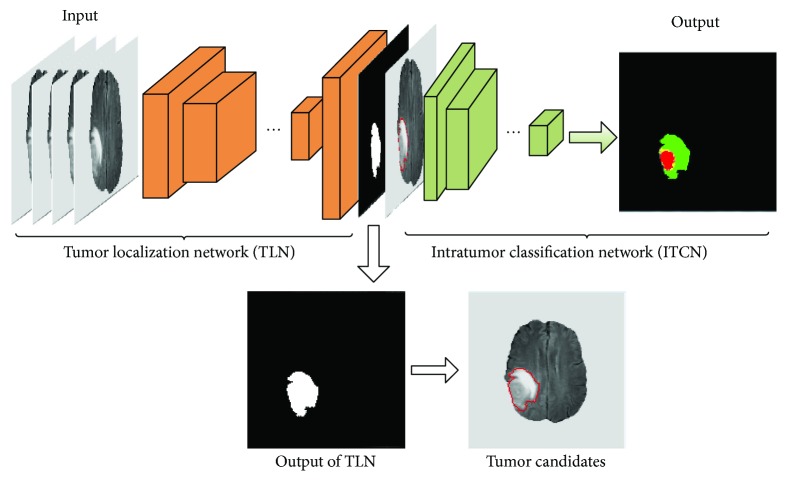
An illustrative overview of the proposed deep cascaded convolutional neural network for a fast and accurate tumor segmentation.

The starting point of the proposed system is *in vivo* MRI data consisting of four different sequences (FLAIR, T1, T1c, and T2), and the endpoint becomes a characterized tumor (see Figure 1). In the output image, a brain tumor is classified into four different zones: necrosis, edema, nonenhancing tumor, and enhancing tumor.

More specifically, the architecture of the proposed system includes an FCN followed by a CNN which accompanies small convolution kernels (see Figure 1). So the segmentation task based on this cascaded network can be divided into two major steps. In the first step, the pixel-wise FCN was used to quickly localize the tumor by marking the tumor region. Then, the patch-wise CNN was used to further categorize the above-identified tumor region into different subregions representing different pathologies. This system design was motivated and justified as follows. First, the FCN can take a whole image as the input and localization of a complete tumor only requires one-pass of the forward propagation. Thus, it can remarkably improve the segmentation efficiency. Second, this combination of FCN and CNN can alleviate the pixel sample class imbalance problem which is serious in MRI images. Thus, it can capture better segmentation details. Third, the intratumor characterization in the second step will only need to be applied to the tumor regions localized in the first step instead of the entire image, thereby significantly reducing forward computing time. Hereafter, the FCN and the CNN are referred as to tumor localization network (TLN) and intratumor classification network (ITCN), respectively.

#### 3.1.1. A Description of TLN

**Figure 2:**
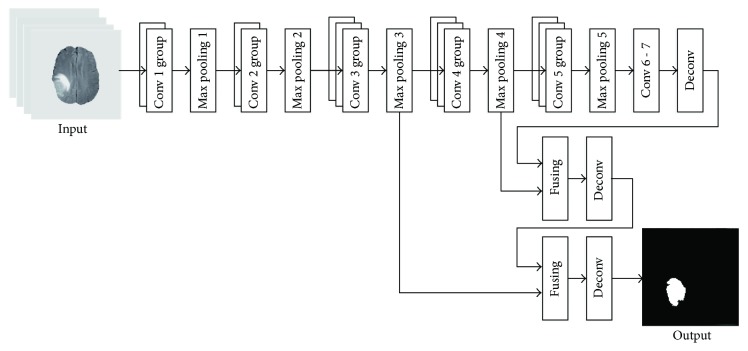
An illustration of the architecture of the TLN subnet for pixel-wise prediction.

We modified the FCN-8s architecture [22] to model our TLN. The input channels (RGB) in the original FCN-8s were changed to 4 channels in order to account for 4 different MRI modalities. And the 21 output channels in the original FCN-8s were changed to 2, corresponding to either the tumor region or the nontumor region. As shown in Figure 2, after the operations of the convolution and pooling, the feature map became smaller in size (see Table 3). To obtain a higher resolution of the final features, the input images (size 240 × 240) were padded to 438 × 438 using zero padding [22]. Additionally, the deconvolution was applied so that the size of output image matched with that of the input image. It is worth noting that multiple convolutional kernels were used in each convolutional layer for a better feature extraction (e.g., edges, curves, and corner).

We observed that a significant amount of low-level feature details such as location and edge could be lost after convolution striding and pooling. However, these lost features were valuable for semantic segmentation. Thus, two skip connections [22] were introduced for two purposes: (1) mitigating the loss of local image features and (2) combining local information obtained from intermediate layers (i.e., max pooling 4 and max pooling 3, resp.) with the global information in these deep layers (i.e., after 7 convolution layers). All relevant parameters used in the subnet TLN are shown in Table 3 below.

#### 3.1.2. A Description of ITCN

**Figure 3:**
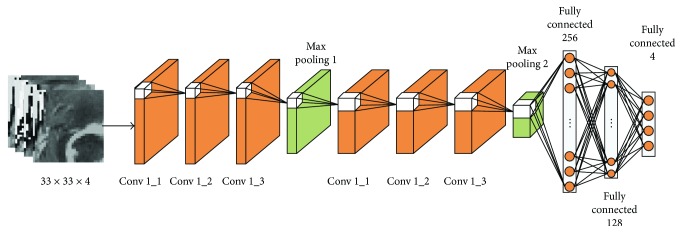
An illustration of the second subnet ITCN for the intratumoral classification. The classification was done in a patch-to-patch fashion.

The proposed ITCN includes two convolutional layer groups (3 layers each), two max pooling layers, and three fully connected layers. Recall that the TLN yields a binary tumor map for a given MRI image and the ITCN (see Figure 3) further classifies the identified tumor into 4 different subregions. Formally, for each location (*i*, *j*) within the identified tumor map, 4 patches (size of 33 × 33) centered on the (*i*, *j*) location were extracted from the original 4 input channels (FLAIR, T1, T1c, and T2) and subsequently used as the input to the ITCN. More details of this ITCN subnet are listed in Table 4.

In the ITCN, as inspired by the work of Simonyan and Zisserman [24], multiple convolutional layers with small kernels (3 × 3 pixels) were used. An alternative approach would be an architecture with fewer layers and larger kernels. Theoretically, two cascaded convolutional layers with two 3 × 3 kernels have similar effects on the receptive fields, as compared to one convolutional layer with a 5 × 5 kernel. But two cascaded layers with two 3 × 3 kernels result in more complex nonlinearities and fewer weights. Fewer weights lead to a less computing cost and can also alleviate the possibility of overfitting. It is generally understood that, with the increase of the CNN's depth, a CNN can gain higher representation capacity. As shown in Figure 3, in each of the two pooling layers, a 3 × 3 overlapping subwindow with a stride of 2 was applied to the feature maps for reducing feature dimension and integrating higher-level features. The detailed hyperparameters of the ITCN can be found in Table 4 below.

### 3.2. Implementation

All numerical experiments were conducted using a Dell workstation equipped with dual Intel E5-2603 CPUs and a middle-end GPU graphic card (GeForce GTX 1080, NVIDIA, CA, USA). The operation system of the workstation is Ubuntu (version 14.04). The proposed cascaded neural network has been implemented using Python (version 2.7) under the framework of Caffe, an open-source deep learning platform (http://caffe.berkeleyvision.org/). Some essential details are discussed below.

#### 3.2.1. Preprocessing

As recommended by the literature [25], MRI data were preprocessed before the proposed cascaded neural network was applied. Basically, the N4ITK method was first used to correct the distortion of MRI data, followed by data normalization.

Given an image *X*, *x*(*i*, *j*) is the intensity corresponding to the *j*th column at the *i*th row of *X*(*i*, *j* = 1, 2,…, 240). The data intensity normalization procedure is briefly described below:

1. Removed the top 1% and bottom 1% from each slice of the MRI data.
2. For each slice of MRI data *X*, a normalized image *X*′ was obtained. In the scaled image *X*′, each intensity value *x*′(*i*, *j*) can be obtained as follows:

(1) $$\begin{array}{c} x^{\prime }\left(i,j\right)=\frac{\left(x\left(i,j\right)-\bar{X}\right)}{X_{s}}, \end{array}$$

where *x*(*i*, *j*) is the gray value of pixel (*i*, *j*) prior to the normalization and $\bar{X}$ and *X*_*s*_ are the mean and standard deviation of the unscaled image *X*, respectively.

**Figure 4:**
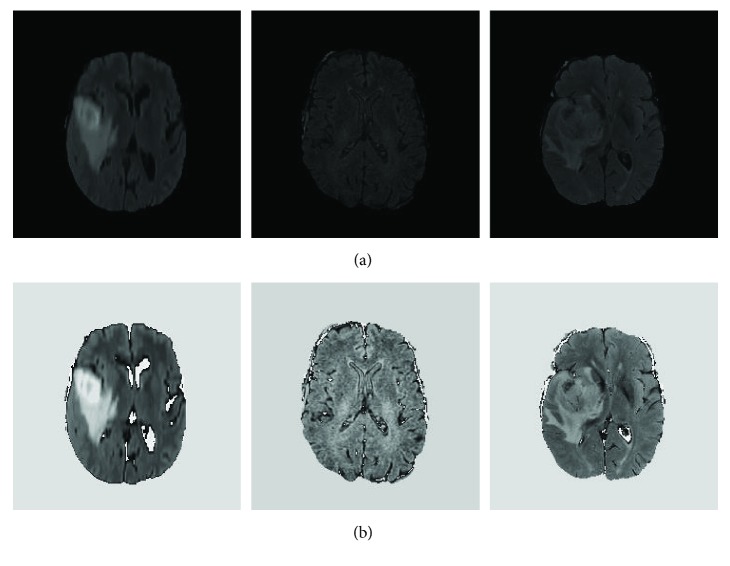
Randomly selected examples of FLAIR slices before (a) and after (b) the above-mentioned intensity normalization.

The above-mentioned preprocessing method was used to process each modality MRI data including FLAIR, T1, T1c, and T2. Particularly, the FLAIR images were generated using fluid-attenuated inversion recovery protocol and useful in terms of differentiating the brain tumor from its normal background.  Figure 4 presents some FLAIR slices before and after using the proposed image intensity normalization. We randomly selected 3 different cases from the FLAIR dataset. As shown in Figure 4 below, it is easy to find that the above-mentioned data normalization can improve the comparability of different slices.

#### 3.2.2. Convolution Operation

Each feature map *Z* shown in Figures 1, 2, and 3 was associated with one convolution kernel. *Z* was computed as follows:

(2) $$\begin{array}{c} Z=b+\overset{k}{\underset{r=1}{\sum }}W_{r}*X_{r}, \end{array}$$

where *k* is the number of input channels, *b* is a bias term, *X*_*r*_ is an image from the *r*th input channel, and *W*_*r*_ is the weight associated with the *r*th channel. In (2), ^∗^ denotes a convolution operator.

#### 3.2.3. Nonlinear Activation Function

In our study, the TLN used rectified linear unit (ReLU) function [23] to perform nonlinear transformations. This selection was because ReLU could achieve better results as compared to the classical sigmoid and hyperbolic tangent functions. The use of ReLU was also able to accelerate the training [26]. Mathematically, the ReLU function is defined below:

(3) $$\begin{array}{c} f\left(z\right)=\max\left(0,z\right). \end{array}$$

In the ITCN, the leaky rectifier linear unit (LReLU) [27] was used. This was because imposing zeros (see (3)) could negatively affect the calculation of gradients. During the training of this neural network, zero gradients will significantly slow down the adjustments of weights. The LReLU function reads

(4) $$\begin{array}{c} f\left(z\right)=\max\left(0,z\right)+\alpha \min\left(0,z\right), \end{array}$$

where *α* is the leakiness parameter [18].

To address the multiclassification problem, a well-known softmax function was used to transform the neural network outputs to probability distributions. Softmax is defined as follows:

(5) $$\begin{array}{c} Y_{i}=soft\max\left(Z_{i}\right)=\frac{e^{Z_{i}}}{\left\|e^{Z}\right\|}, \end{array}$$

where *Z*_*i*_ is the output from the *i*th neuron and *Y*_*i*_ is the probability of input pixel corresponding to the *i*th class. In the TLN, *i* = 1 or 2 because the TLN was to perform a binary classification in the first step. In the ITCN, *i* = 1, 2, 3, 4 since the ITCN was to classify the MRI data into four classes.

#### 3.2.4. Loss Function

Given a set of weights of the proposed neural network *θ*, a categorical cross-entropy loss function was used to compute the loss of ground truth and predicted probability distribution. Mathematically, under an arbitrary prediction for the *i*th pixel, the predition loss can be defined as

(6) $$\begin{array}{c} L\left(\theta \right)=-\overset{C}{\underset{j=1}{\sum }}Y_{ij}^{\prime }\log\left(Y_{ij}\right), \end{array}$$

where **Y**′, *Y*, and *C* are a one-hot vector, the predicted probability distribution, and the number of classes, respectively.

In the TLN, predictions were made for each pixel of the input image so that the loss function can be written as follows:

(7) $$\begin{array}{c} L\left(\theta^{\prime }\right)=-\frac{1}{S}\overset{S}{\underset{i=1}{\sum }}\overset{C}{\underset{j=1}{\sum }}Y_{ij}^{\prime }\log\left(Y_{ij}\right), \end{array}$$

where *C* = 2 and *S* is the pixel number of the input image. In every training, only one input image was used (the size of minibatch was 1).

Now referring to the ITCN, the loss function was calculated in conjunction with the concept of mini-batch. Thus, the loss function has the following form,

(8) $$\begin{array}{c} L\left(\theta^{\prime \prime }\right)=-\frac{1}{M}\overset{M}{\underset{i=1}{\sum }}\overset{C}{\underset{j=1}{\sum }}Y_{ij}^{\prime }\log\left(Y_{ij}\right), \end{array}$$

where *C* = 4 and *M* is the size of minibatch. Of note, in this study, *M* = 256.

To achieve better generation ability and avoid overfitting, L2 regularization terms were also added to (7) and (8). Thus, the final forms of the loss functions are

(9) $$\begin{array}{c} L\left(\theta^{\prime }\right)=-\frac{1}{S}\overset{S}{\underset{i=1}{\sum }}\overset{C}{\underset{j=1}{\sum }}Y_{ij}^{\prime }\log\left(Y_{ij}\right)+\frac{\lambda }{S}\overset{Q}{\underset{k=1}{\sum }}\left|{\theta_{k}^{\prime }}^{2}\right|, \end{array}$$

(10) $$\begin{array}{c} L\left(\theta^{\prime \prime }\right)=-\frac{1}{M}\overset{M}{\underset{i=1}{\sum }}\overset{C}{\underset{j=1}{\sum }}Y_{ij}^{\prime }\log\left(Y_{ij}\right)+\frac{\lambda }{M}\overset{Q}{\underset{k=1}{\sum }}\left|{\theta_{k}^{\prime }}^{2}\right|, \end{array}$$

where *λ* is a regularization constant and *Q* is the number of model parameter.

#### 3.2.5. Optimization Method

Equations (9) and (10) were minimized using the minibatch stochastic gradient descent (SGD) algorithm. To avoid numerical oscillations and accelerate convergence, the momentum method [23] was used. This process can be described as iterations from (11) to (13).

(11) $$\begin{array}{c} g_{t}=\nabla_{t-1}L\left(\theta_{t-1}\right), \end{array}$$

(12) $$\begin{array}{c} m_{t}=\mu *m_{t-1}-\eta_{t}g_{t}, \end{array}$$

(13) $$\begin{array}{c} \theta_{t}=\theta_{t-1}+m_{t}. \end{array}$$

In (11), (12), and (13), the subscript *t* is the iteration number and *θ* corresponds to *θ*′ in (9) or *θ*^″^ in (10). *L*(*θ*_*t*−1_) is the loss function when a parameter set *θ*_*t*−1_ is used. *g*_*t*_, *m*_*t*_, and *μ* are the gradient, momentum, and momentum coefficient, respectively. We set *μ* = 0.99 and *μ* = 0.9 in the TLN and ITCN, respectively. Here, *η*_*t*_ is the learning rate.

To suppress the SGD noise and guarantee convergence, the learning rate *η*_*t*_ attenuates linearly from the initial learning rate *η*_0_ to the final learning rate *η*_*τ*_ as the iteration progresses:

(14) $$\begin{array}{c} \eta_{t}=\left(1-\gamma \right)\eta_{0}+{\gamma \eta }_{\tau }, \end{array}$$

(15) $$\begin{array}{c} \gamma =\frac{t}{\tau }, \end{array}$$

where *τ* is the total iteration number. In this study, we set *η*_*τ*_ = *η*_0_/100.

#### 3.2.6. Training Details

The initial and final learning rates of the TLN model were set to 1*e*−8 and 1*e*−10, respectively. The total iteration *τ* = 2*e*6, and the momentum coefficient was 0.99. In the ITCN subnet, the initial and final learning rates were set to 1*e*−3 and 1*e*−5, respectively. In the ITCN subnet, the total iteration *τ* = 2*e*6 and the momentum coefficient *μ* = 0.9.

During the training of the TLN subnet, we used the transfer learning technique [28, 29]. The initial weights were obtained from a pretrained model that was trained using ImageNet in [24]. But initial weights of the 4th input channel were initialized using the average of the original 3 input channel (RGB) weights. And the final two output channels were initialized with the Xavier method [30]. Then, fine-tuning of the TLN was performed by the optimization process described above ((11), (12), and (13)) using the MRI training data. However, the training of the ITCN subnet was started from scratch and the weights were initialized with the Xavier method [30]. To avoid overfitting, we used the dropout regularization [31] and the dropout ratio was set to 0.5 in all fully connected layers. Weight decay was set as 0.005.

### 3.3. Datasets and Evaluation Metrics

In order to train and evaluate the proposed system, numerical experiments were carried out using *in vivo* human patient data provided by the BRATS 2015 database [32]. The BRATS 2015 database contains 220 HGG and 54 LGG. Experimental data have been labeled, and five labels were used: normal brain tissues (noncancerous zone), necrosis, edema, nonenhancing tumor, and enhancing tumor. These pixel-wise delineations were considered the ground truth in this study. Each case contains four sequences of MRI data, namely, T1, T1c, T2, and FLAIR. The dimension of each MRI modality is 155 × 240 × 240 (slice number × length × width). All MRI data were spatially registered and stored as signed 16-bit integers. But only positive values were used.

The tenfold crossvalidation method [33] was used to evaluate the proposed system. More specifically, the 274 cases were divided into a training set (240 cases) and a testing set (34 cases). The 240 training cases were equally divided into 10 subsets in which 9 subsets were used as the training and 1 subset was used as the validation. In the training phase of the TLN subnet, all subregions within a tumor were merged into one tumor region. Thus, in the binary ground truth, zero represents the noncancerous tissues while one represents cancerous regions. In the training phase of the ITCN subnet, we randomly selected 4,700,000 image patches (33 × 33) from the training set, which correspond to 1,175,000 patches for each label (4 different classes).

The quantitative evaluations were conducted for 3 different tumor regions: complete tumor region (including all four tumor subregions), core tumor region (including all tumor structures except edema), and enhancing tumor region (only including the enhanced tumor structure). For each type of regions, we compute DSC [34], PPV, and sensitivity [35] as quantitative evaluation metrics.

DSC measures the overlap between the ground truth and the automatic segmentation. It is defined as

(16) $$\begin{array}{c} DSC=\frac{P_{1}\cap T_{1}}{\left(P_{1}+T_{1}\right)/2}, \end{array}$$

where *P*_1_ and *T*_1_ represent the positive values of the model prediction and the ground truth, respectively.

PPV is the proportion of the true positive in all segmentation tumor points. It is defined as

(17) $$\begin{array}{c} PPV=\frac{P_{1}\cap T_{1}}{P_{1}}. \end{array}$$

Sensitivity is the proportion of the detected tumor points in all ground truth tumor points. It is defined as

(18) $$\begin{array}{c} Sensitivity=\frac{P_{1}\cap T_{1}}{T_{1}}. \end{array}$$

The proposed system was compared with some other published methods. Those methods all have been validated on the BRATS 2015 dataset. A one-step segmentation method based on the FCN-8s was also implemented for the purpose of comparison. The FCN-8s can segment the input MRI images into 5 classes in a single step.

## 4. Results

### 4.1. Qualitative Observations

**Figure 5:**
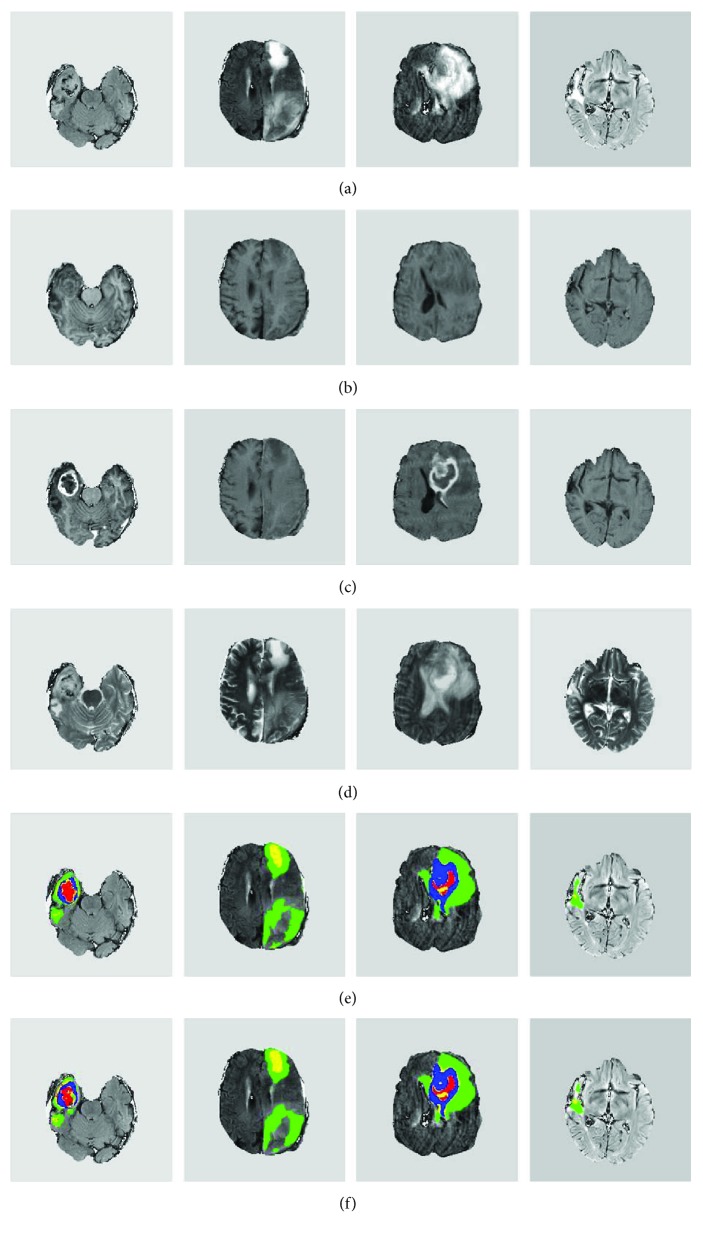
Representative examples of computer segmentation results of four brain tumors. (a–d) The original FLAIR, T1, T1c, and T2 slices, respectively. (e) The ground truth overlaid with the FLAIR image. (f) Segmentation results overlaid with the FLAIR image. (e, f) Red, green, yellow, and blue colors denote necrosis, edema, nonenhancing tumor, and enhancing tumor, respectively.

Overall, we found that the proposed system can accurately delineate gliomas. Visual inspections were conducted for testing data to validate the segmentation results of our proposed method.  Figure 5 shows four selected examples. It can be observed that our method can effectively localize and segment brain tumors with vastly different shapes and sizes. Visually, the computer segmentation is comparable to the ground truth.

**Figure 6:**
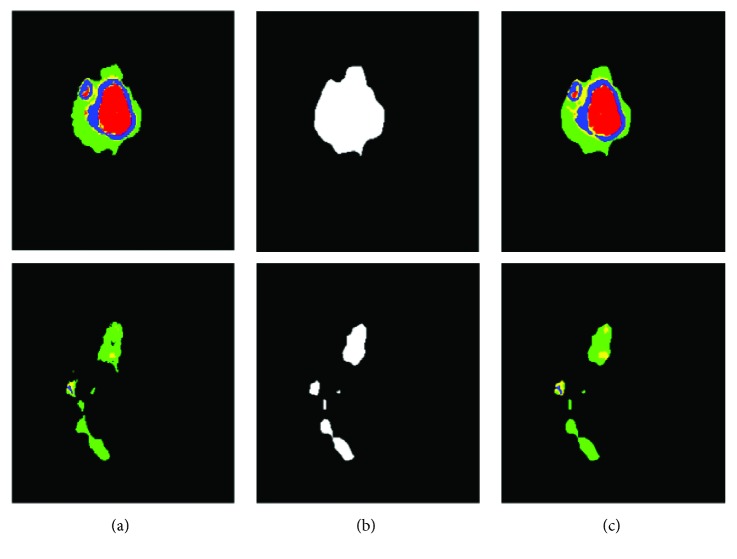
Two slices of computer segmentation result in a testing case: (a–c) the ground truth, results of tumor localization using the TLN subnet, and the intratumor segmentation results using the ITCN subnet, respectively. (a, c) Red, green, yellow, and blue colors denote necrosis, edema, nonenhancing tumor, and enhancing tumor, respectively.

Also, the proposed system led to good details around boundaries.  Figure 6 presents two representative examples of this observation. Since these brain tumors are complex, Figure 6 shows some good showcase examples. During the process, we found that the TLN subnet was able to effectively identify nearly all the tumor pixels. Subsequently, the ITCN subnet efficiently classified the tumor region into four subregions. Our method could largely detect the complete tumor and classify it to different tumor subregions from multimodality MRI images though there were a few misclassifications. This is not surprising because, pathologically, the brain glioma tumors invade their surrounding tissues rather than displacing them. Hence, the appearance of cancerous tissues and their surrounding (normal) tissues could be fairly similar under MRI.

**Figure 7:**
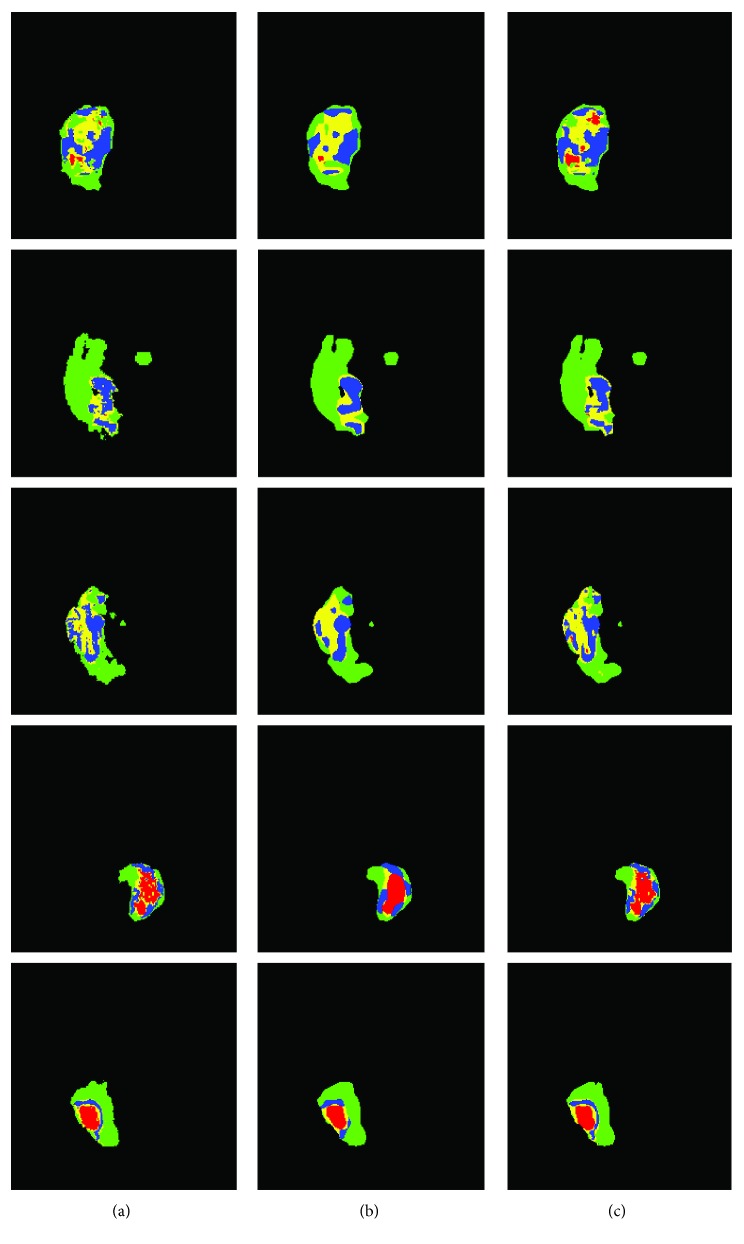
Examples of segmentation results from five typical slices comparing the FCN-8s (b) and the proposed method (c). (a) The ground truth. In this figure, red, green, yellow, and blue colors denote necrosis, edema, nonenhancing tumor, and enhancing tumor, respectively.

We also found that, as compared to the FCN-8s with one-step segmentation, the proposed system could segment heterogeneous gliomas with a better boundary detail. The results of the proposed method and FCN-8s are compared in Figure 7. Five different typical slices representing significantly different tumor shapes and sizes are shown in this figure. It is easy to see that the results obtained from the proposed method (the third column) are more similar to the ground truth (the first column), as compared to the classification results by the FCN-8s (the second column). Furthermore, boundaries of various subregions obtained by the FCN-8s were overly smoothed and, perhaps, inaccurate. But our method using the ITCN had better boundaries of the enhancing and nonenhancing regions.

### 4.2. Evaluation and Comparison

The quantitative comparisons with other methods in terms of DSC are summarized in Tables 5 and 6. All experiments were conducted on the BRATS 2015 dataset. The results of Table 5 were obtained by using the combined testing set of HGG and LGG, whereas results shown in Table 6 only used HGG data.

Obviously, the proposed cascaded neural network obtains the comparable and better DSC value on all tumor regions. Based on the combined testing dataset (see Table 5), our method obtained better comprehensive performance values (0.89, 0.77, and 0.80) as compared to other methods. Although the method proposed by Kamnitsas et al. [21] yields a slightly higher DSC value in the complete tumor, they obtained lower DSC values in core tumor and enhancing tumor. Actually, in their work, a 3D CNN and the structure prediction technology were adopted (i.e., conditional random field). Thus, it is computationally time-consuming and needs extra postprocessing. Furthermore, the method proposed by Dong et al. [36] yielded a slightly higher DSC value in core tumor and Yi et al. [37] yielded the same DSC value in enhancing tumor.

As can be seen in Table 6, based on the HGG testing dataset, our method obtained the highest DSC values in the complete tumor and enhancing tumor categories. Although the method proposed by Dong et al. [36] yielded a higher DSC value in the core tumor cases, it obtained a lower DSC value in the complete tumor category.

Recently, we found that Pereira et al. [39] also proposed a hierarchical brain tumor segmentation approach from MRI HGG images. The difference between their method and our method is that they adopted the FCN in both first and second steps. We compared the results of our method with their method (see Table 7). Our proposed approach obtained the better DSC values (0.90, 0.81, and 0.81) in all tumor regions. Furthermore, the proposed method also yielded higher PPV values in the complete and enhancing tumor categories and a higher sensitivity in the core tumor category. Of note, Pereira et al. [39] trained and tested on the BRATS 2013 dataset but we on the BRATS 2015 dataset.

Additionally, the segmentation speed for testing data was also documented (see Table 8). Computational performance of the first four methods was obtained through respective publications [18, 19, 21, 36]. The proposed method is efficient as compared to other methods. It only takes averagely 1.54 seconds in order to segment a slice and only runs slightly slower than the FCN-8s (0.98 seconds). This is understandable because the proposed method needs two-stage segmentation while the FCN-8s only needs a forward computation. However, the FCN-8s yields less accurate and overly smooth boundary maps. Of note, adopting the FCN for image semantic segmentation is faster than the traditional method based on patch-wise [22, 36]; despite computational efficiency, tests reported in the literature were done using slightly different computing platforms.

## 5. Discussions and Conclusions

In this work, a cascaded neural network was designed, implemented, and tested. The proposed system consists of two steps. In the first step, the TLN subnet was used to localize the brain tumor. Then, the ITCN subnet was applied to the identified tumor regions to further classify the tumor into four subregions. We also adopted the advanced technologies to train and optimize the proposed cascaded neural network. Numerical experiments were conducted on 274 patient *in vivo* data sets from the BRATS 2015. DSC, PPV, and sensitivity were used as metrics for segmentation accuracy.

Based on quantitative and qualitative evaluations, we found that the proposed approach was able to accurately localize and segment complex brain tumors. We stipulate that there are two reasons. First, the ITCN subnet only represents and subsequently classifies the intratumoral region whereas other methods need to represent and classify all heterogeneous brain tissues. Second, intratumor subregions are usually very small proportions of the entire image. Other neural networks (e.g., FCN-8s) may suffer from the imbalance of different pixel labels. In the TLN subnet, our proposed method merged different tumor subregions into a whole tumor. Thus, the imbalance can be somewhat mitigated. In the ITCN subnet, we adopted the same quantity image patches of each class to train and optimize the model. In the future, deep learning neural networks could be expanded to include histological data and other data to further improve clinical management of brain cancers [40].

Furthermore, the proposed cascaded neural network can, on average, complete a segmentation task within 1.54 seconds. The proposed TLN subset only requires a forward computation for localizing the whole tumor region in the first step. Then, the ITCN subnet only needs to classify tumor candidate pixels into different class subregions within a much-reduced region located by the TLN, thereby improving the computing efficiency.

## Figures and Tables

**Table 1:** A summary of brain tumor segmentation methods based on traditional machine learning. Only methods using MRI data were included in this table.

Number	Publication	Database	Summary of method	Performance	
1	Corso et al. [5]	20 cases of *in vivo* brain tumors; T1, T1-C, T2, FLAIR	A hybrid method combining an affinity-based segmentation method with a generative model	0.62–0.69 (Jaccard)	
2	Hamamci et al. [6]	Synthetic data from Utah + *in vivo* data from Harvard	A cellular automata method combining a probability framework	0.72 (DICE complete tumor)	
3	Mehmood et al. [7]	BrainWeb data + *in vivo* brain tumors; T1, T1-weighted, T2, T2-weighted	A novel saliency model for lesion localization and an N-cut graph segmentation model for classification	83%~95% (classification accuracy)	
4	Havaei et al. [8]	MICCAI-BRATS 2013 dataset	Hand-crafted features + a support vector machine	0.86 (DICE complete tumor)	
5	Usman and Rajpoot [9]	MICCAI-BRATS 2013 dataset	Automated wavelet-based features + a random forest classifier	0.88 (DICE complete tumor)	
6	Tustison et al. [10]	MICCAI-BRATS 2013 dataset	Combine a random forest model with a framework of regularized probabilistic segmentation	0.88 (DICE complete tumor)	
7	Zikic et al. [11]	40 multichannel MR images, including DTI	Decision forests using context-aware spatial features for automatic segmentation of high-grade gliomas	GT: 0.89 NE: 0.70	AC: 0.84 E: 0.72
				(10/30 tests)	
8	Pinto et al. [12]	MICCAI-BRATS 2013 dataset	Using appearance- and context-based features to feed an extremely randomized forest	0.83 (DICE complete tumor)	
9	Bauer et al. [13]	10 multispectral patient datasets	Combines support vector machine classification with conditional random fields	GT: 0.84 AC: 0.84	NE: 0.70 E: 0.72
				(Intrapatient regularized)	

**Table 2:** A summary of brain tumor segmentation methods based on deep-learning neural networks. Only methods using MRI data were included in this table.

Number	Publication	Database	Summary of method	Performance (DICE)		
				Complete	Core	Enh
1	Urban et al. [14]	MICCAI-BRATS 2013 dataset	3D CNN with 3D convolutional kernels	0.87	0.77	0.73
2	Zikic et al. [15]	MICCAI-BRATS 2013 dataset	Apply a CNN in a sliding-window fashion in the 3D space	0.84	0.74	0.69
3	Davy et al. [16]	MICCAI-BRATS 2013 dataset	A CNN with two pathways of both local and global information	0.85	0.74	0.68
4	Dvorak and Menze [17]	MICCAI-BRATS 2013 dataset	Structured prediction was used together with a CNN	0.83	0.75	0.77
5	Pereira et al. [18]	MICCAI-BRATS 2013 dataset	A CNN with small 3 × 3 kernels	0.88	0.83	0.77
6	Havaei et al. [19]	MICCAI-BRATS 2013 dataset	A cascade neural network architecture in which “the output of a basic CNN is treated as an additional source of information for a subsequent CNN”	0.88	0.79	0.73
7	Lyksborg et al. [20]	MICCAI-BRATS 2014 dataset	An ensemble of 2D convolutional neural networks +doing a volumetric segmentation by three steps	0.80	0.64	0.59
8	Kamnitsas et al. [21]	MICCAI-BRATS 2015 dataset	Using 3D CNN, two-scale extracted feature, 3D dense CRF as postprocessing	0.85	0.67	0.63

**Table 3:** Parameters used in the subnet TLN. In each convolutional layer, the feature maps had been padded by 1 prior to the convolution so that all intermediate feature maps do not change their sizes before and after the convolution.

Number	Layer name	Filter size	Stride	Number of Filters	Output
1	Conv 1_1 + ReLU	3^∗^3	1	64	438^∗^438^∗^64
2	Conv 1_2 + ReLU	3^∗^3	1	64	438^∗^438^∗^64
3	Max pooling 1	2^∗^2	2	—	219^∗^219^∗^64
4	Conv 2_1 + ReLU	3^∗^3	1	128	219^∗^219^∗^128
5	Conv 2_2 + ReLU	3^∗^3	1	128	219^∗^219^∗^128
6	Max pooling 2	2^∗^2	2	—	110^∗^110^∗^128
7	Conv 3_1 + ReLU	3^∗^3	1	256	110^∗^110^∗^256
8	Conv 3_2 + ReLU	3^∗^3	1	256	110^∗^110^∗^256
9	Conv 3_3 + ReLU	3^∗^3	1	256	110^∗^110^∗^256
10	Max pooling 3	2^∗^2	2	—	55^∗^55^∗^256
11	Conv 4_1 + ReLU	3^∗^3	1	512	55^∗^55^∗^512
12	Conv 4_2 + ReLU	3^∗^3	1	512	55^∗^55^∗^512
13	Conv 4_3 + ReLU	3^∗^3	1	512	55^∗^55^∗^512
14	Max pooling 4	2^∗^2	2	—	28^∗^28^∗^512
15	Conv 5_1 + ReLU	3^∗^3	1	512	28^∗^28^∗^512
16	Conv 5_2 + ReLU	3^∗^3	1	512	28^∗^28^∗^512
17	Conv 5_3 + ReLU	3^∗^3	1	512	28^∗^28^∗^512
18	Max pooling 5	2^∗^2	2	—	14^∗^14^∗^512
19	Conv 6 + ReLU	7^∗^7	1	4096	8^∗^8^∗^4096
20	Conv 7 + ReLU	1^∗^1	1	4096	8^∗^8^∗^4096

**Table 4:** A list of parameters used in the proposed subnet ITCN. In each convolutional layer, the feature maps had been padded by 1 prior to the convolution so that the convolution do not change the size of the resultant feature map.

Number	Layer name	Filter size	Stride	Number of filters	FC units	Output
1	Conv 1_1 + LReLU	3^∗^3	1	64	—	33^∗^33^∗^64
2	Conv 1_2 + LReLU	3^∗^3	1	64	—	33^∗^33^∗^64
3	Conv 1_3 + LReLU	3^∗^3	1	64	—	33^∗^33^∗^64
4	Max pooling 1	3^∗^3	2	—	—	16^∗^16^∗^64
5	Conv 2_1 + LReLU	3^∗^3	1	128	—	16^∗^16^∗^128
6	Conv 2_2 + LReLU	3^∗^3	1	128	—	16^∗^16^∗^128
7	Conv 2_3 + LReLU	3^∗^3	1	128	—	16^∗^16^∗^128
8	Max pooling 2	3^∗^3	2	—	—	8^∗^8^∗^128
9	FC1 + dropout	—	—	—	8192	256
10	FC2 + dropout	—	—	—	256	128
11	FC3 + softmax	—	—	—	128	4

**Table 5:** A summary of DSC quantitative comparison on BRATS 2015 combined dataset (HGG and LGG).

Method	Dataset	Grade	DSC		
			Complete	Core	Enh
Pereira et al. [38]	BRATS 2015 Challenge	Combined	0.78	0.65	0.75
	BRATS 2015 Training	Combined	0.87	0.73	0.68
Havaei et al. [19]	BRATS 2015 Challenge	Combined	0.79	0.58	0.69
Kamnitsas et al. [21]	BRATS 2015 Challenge	Combined	0.85	0.67	0.63
	BRATS 2015 Training	Combined	*0.90*	0.76	0.73
Dong et al. [36]	BRATS 2015 Training	Combined	0.86	*0.86*	0.65
Yi et al. [37]	BRATS 2015 Training	Combined	0.89	0.76	*0.80*
FCN-8s	BRATS 2015 Training	Combined	0.84	0.71	0.63
Proposed	BRATS 2015 Training	Combined	0.89	0.77	*0.80*

**Table 6:** A summary of DSC quantitative comparison on BRATS 2015 HGG dataset.

Method	Dataset	Grade	DSC		
			Complete	Core	Enh
Pereira et al. [38]	BRATS 2015 Training	HGG	0.87	0.75	0.75
Havaei et al. [19]	BRATS 2015 Challenge	HGG	—	—	—
Kamnitsas et al. [21]	BRATS 2015 Training	HGG	—	—	—
Dong et al. [36]	BRATS 2015 Training	HGG	0.88	*0.87*	*0.81*
Yi et al. [37]	BRATS 2015 Training	HGG	0.89	0.79	0.80
FCN-8s	BRATS 2015 Training	HGG	0.88	0.76	0.71
Proposed	BRATS 2015 Training	HGG	*0.90*	0.81	*0.81*

**Table 7:** A comparison of our proposed method with hierarchical brain tumor segmentation [39] on DSC, PPV, and sensitivity metrics.

Method	DSC			PPV			Sensitivity		
	Complete	Core	Enh	Complete	Core	Enh	Complete	Core	Enh
Pereira et al. [39]	0.85	0.76	0.74	0.80	*0.78*	0.74	*0.92*	0.79	*0.78*
Proposed	*0.90*	*0.81*	*0.81*	*0.91*	0.77	*0.87*	0.87	*0.84*	0.76

**Table 8:** Comparisons of segmentation time among six different methods. The estimation of time for the proposed method was based on the acceleration of GPU.

Method	Time
Pereira et al. [18]	8 s–24 min
Havaei et al. [19]	8 min
Kamnitsas et al. [21]	30 s
Dong et al. [36]	2-3 s
FCN-8s	0.98 s
Proposed	1.54 s
